# Retraction: Suppression of MMP-2 attenuates TNF-α induced NF-κB activation and leads to JNK mediated cell death in glioma

**DOI:** 10.1371/journal.pone.0328090

**Published:** 2025-07-11

**Authors:** The PLOS One Editors

After the publication of this article [1], concerns were raised with results presented in Figs 1, 2, 7, 8, and S1. Specifically,

- The following panels appear similar:
  - Fig 1C 4910 MMP-2 and Fig 1C 5310 MMP-2
  - Fig 1C 4910 GAPDH and Fig 1C 5310 GAPDH
  - Fig S1 4910 SP600125 TUNEL and Fig S1 5310 pSV TUNEL
  - Fig 7B 4910 Fas-L and Fig 7B 5310 Fas-L
  - Fig 7B 4910 JNK and Fig 7B 5310 JNK, when flipped horizontally
  - Lanes 2-5 of Fig 7C 4910 p-JNK and lanes 2-5 of Fig 7C 5310 p-JNK, when levels are altered
- There appear to be irregularities in the background of the following panels:
  - Fig 2C (right) Casp-3
  - Fig 7B 4910 FADD
- The Fig 8D 5310 Mock panel appears to partially overlap with the Fig 8D pSV panel

The authors did not respond to the journal’s communications.

In light of the above concerns, which call into question the reliability and integrity of the published image data and their associated quantified results, the *PLOS One* Editors retract this article.

All authors either did not respond directly or could not be reached.
